# Prognostic value of the extent of resection in supratentorial WHO grade II astrocytomas stratified for IDH1 mutation status: a single-center volumetric analysis

**DOI:** 10.1007/s11060-016-2177-y

**Published:** 2016-06-25

**Authors:** Christine Jungk, Moritz Scherer, Andreas Mock, David Capper, Alexander Radbruch, Andreas von Deimling, Martin Bendszus, Christel Herold-Mende, Andreas Unterberg

**Affiliations:** 1 Department of Neurosurgery, Heidelberg University Hospital, Im Neuenheimer Feld 400, 69120 Heidelberg, Germany; 2 Division of Neuropathology, Institute of Pathology, Heidelberg University Hospital, Heidelberg, Germany; 3 Division of Neuroradiology, Department of Neurology, Heidelberg University Hospital, Heidelberg, Germany; 4 German Cancer Consortium (DKTK), CCU Neuropathology, German Cancer Research Center (DKFZ), Heidelberg, Germany

**Keywords:** Low-grade astrocytoma, Volumetric analysis, Extent of resection, IDH1, Intraoperative MRI, Survival

## Abstract

Current evidence supports a maximized extent of resection (EOR) in low-grade gliomas (LGG), regardless of different histological subtypes and molecular markers. We therefore evaluated the prognostic impact of extensive, mainly intraoperative (i)MRI-guided surgery in low-grade astrocytomas stratified for IDH1 mutation status. Retrospective assessment of 46 consecutive cases of newly diagnosed supratentorial WHO grade II astrocytomas treated during the last decade was performed. IDH1 mutation status was obtained for all patients. Volumetric analysis of tumor volumes was performed pre-, intra-, early postoperatively and at first follow-up. Survival analysis was conducted with uni-and multivariate regression models implementing clinical parameters and continuous volumetric variables. Median EOR was 90.4 % (range 17.5–100 %) and was increased to 94.9 % (range 34.8–100 %) in iMRI-guided resections (n = 33). A greater EOR was prognostic for increased progression-free survival (HR 0.23, p = 0.031) and time to re-intervention (TTR) (HR 0.23, p = 0.03). In IDH1 mutant patients, smaller residual tumor volumes were associated with increased TTR (HR 1.01, p = 0.03). IDH1 mutation (38/46 cases) was an independent positive prognosticator for overall survival (OS) in multivariate analysis (HR 0.09, p = 0.002), while extensive surgery had limited impact upon OS. In a subgroup of patients with ≥40 % EOR (n = 39), however, initial and residual tumor volumes were prognostic for OS (HR 1.03, p = 0.005 and HR 1.08, p = 0.007, respectively), persistent to adjustment for IDH1. No association between EOR and neurologic morbidity was found. In this analysis of low-grade astrocytomas stratified for IDH1, extensive tumor resections were prognostic for progression and TTR and, in patients with ≥40 % EOR, for OS.

**Electronic supplementary material:**

The online version of this article (doi:10.1007/s11060-016-2177-y) contains supplementary material, which is available to authorized users.

## Introduction

Low-grade gliomas (LGGs) represent up to 15 % of all primary brain tumors, show a slow but steady growth and hold a better prognosis compared to their high-grade counterparts. Nonetheless, their infiltrative behavior into the surrounding brain parenchyma imposes a tremendous therapeutic challenge. Alongside demographic factors, studies have identified preoperative tumor burden and extent of resection (EOR) as prognosticators of overall survival (OS), progression-free (PFS), or malignant progression-free survival (MPFS) [1–6]. This puts extensive surgery in favor for first-line therapy of resectable LGGs, also in case of recurrence [1–5, 7–9]. However, achieving a radical but safe tumor resection remains a tightrope walk. In this context, intraoperative magnetic resonance imaging (iMRI) offers unique possibilities for intraoperative tumor visualization with a high potential to augment EOR [5, 10–12].

A critical limitation of previous outcome studies in LGG surgery is the analysis of survival regardless of different histological subtypes. A pooled analysis of astrocytomas, oligodendrogliomas and oligoastrocytomas underrates the fact that histology per se confers divergent patient survival. In fact, WHO grade II astrocytomas show an OS of 5–10 years, whereas oligodendrogliomas are expected to live up to 5 years longer [13]. Moreover, molecular characteristics have recently been shown to refine histological subtypes and determine outcome [14–16]. In particular, mutations in the isocitrate dehydrogenase 1 (IDH1) encoding gene which are present in 70–80 % of LGGs confer a favorable outcome in astrocytomas [17] and discriminate lower-grade tumors with a rather benign clinical course from IDH1 wildtype (wt) tumors, that molecularly and clinically behave like glioblastoma [18]. A paradigm shift from a histopathologic towards an integrated molecular classification of gliomas has ultimately led to a recent update of the WHO classification [14, 15, 17–19]. Consequently, when assessing benefits of therapeutic interventions such as extensive surgery, stratification for histological subtypes and molecular markers is mandatory and facilitates comparability of results.

Apart from that, many surgical outcome studies relied on the surgeon’s intraoperative impression or gross estimation of EOR instead of objective volumetric measurements of residual tumor, which seems increasingly inappropriate given the growing importance of EOR in modern glioma surgery.

As a lesson learned from the shortcomings discussed above, we aimed to evaluate the impact of extensive, predominantly iMRI-guided surgery on patient outcome in a histologically well-defined cohort of newly diagnosed, adult supratentorial WHO grade II astrocytomas eligible for tumor resection. In all cases, stratification for IDH1 was performed and EOR was determined objectively by volumetric analysis pre-, intra- and postoperatively.

## Patients and methods

### Patient cohort

Out of a consecutive series of 101 adult supratentorial WHO grade II gliomas treated by micro-neurosurgical tumor resection at the Department of Neurosurgery, University Hospital Heidelberg (Germany) from 2004 to 2013, 60 patients with pure astrocytic tumors were identified. Only patients with first tumor resection at our department were included into survival analysis (n = 46). Retrospective patient selection was limited to common availability of digital perioperative imaging data since 2004, to allow for volumetric analysis.

Medical charts review was performed including clinical parameters [gender, age at first diagnosis, neurologic deficits, Karnofsky Performance Score (KPS)], tumor location (side, lobe, eloquence) and treatment history (resection, radiation, chemotherapy). Only brain regions directly associated with motor or speech function were regarded eloquent.

Approval from the ethics committee of the University of Heidelberg Medical School was obtained prior to conduction of this retrospective study (reference S-327/2014, as of 07-03-2014).

### Histopathologic review

Histopathologic review confirmed all cases as WHO grade II astrocytomas according to the 2007 WHO classification [13]. In case of uncertainty, 1p/19q deletion was excluded by analysis of genome wide DNA copy number changes as previously described [20]. IDH1 mutation status for codon R132H was obtained for all cases by immunohistochemistry [21] or direct sequencing of the mutation hotspot region [22].

### Volumetric analysis of EOR

Routine MRI was evaluated at up to four time points in this study: preoperatively, early postoperatively (<72 h after surgery), at first follow-up (on average 3 months after surgery) and intraoperatively, when applicable (33/46 cases; 72 %). Imaging sequences contained standard T2-, FLAIR- and T1-weighted sequences before and after administration of paramagnetic intravenous contrast agent (gadolinium, 0,1 mmol/kg body weight, single-shot). IMRI was performed at 0.2 T (T) for all procedures until 06/2009 and at 1.5 T ever since. Pre- and postoperative MRI was performed at a field strength of 1.5–3 T.

For volumetric analysis, semi-automatic across-slice segmentation with manual correction was performed based on signal abnormality on T2/FLAIR-weighted images in cm^3^. Segmentation and volumetric calculation was performed with 3D-Slicer Software on axial slices with adjustment on coronal and sagittal planes, respectively [23]. EOR was termed complete (EOR 100 %) if no T2/FLAIR hyperintense residual tumor was detected on postoperative imaging. The difference between preoperative tumor volumes on T2- and T1-weighted imaging (dT2T1) was calculated as an imaging surrogate for infiltrative tumor growth as proposed by Skrap et al. [24]. Volumetric data was regarded as a continuous variable in outcome analysis.

### Outcome analysis

Outcome parameters were OS, PFS, MPFS and time to re-intervention (TTR). OS was defined as time from first image diagnosis until death or last follow-up. PFS was defined as time from first histological diagnosis to radiologic signs of progression or malignization based on the MacDonald/RANO criteria, or death [25–27]. MPFS was defined as time from first histological diagnosis to radiographic signs of malignization [new and treatment-unrelated contrast enhancement (CE) on follow-up MRI], altered histological diagnosis or death. Time after initial treatment to any tumor specific re-intervention (surgery, chemotherapy, radiotherapy) was termed TTR. Median follow-up at the end of the study (November 30, 2015) was 70.3 months (range 17.5–164.6 m).

Analysis was performed for the full study sample (n = 46), for IDH1 mutant (mt) patients (n = 38), for all patients with ≥40 % EOR (n = 39) and for IDH1 mt patients with ≥40 % EOR (n = 32). Thereby, we sought to minimize molecular and surgical bias by adjustment for different anatomical (tumor size, tumor location, intended partial or complete resection) and technical (iMRI) prerequisites for surgery in this retrospective setting. For subgroup analysis, a 40 % EOR cut-off, based on a previous large volumetric LGG study that failed to demonstrate any survival benefit below 40 % EOR [1–6], sought to discriminate between extended biopsies (<40 % EOR) and tumor resections (≥40 % EOR).

### Statistical analysis

Survival associations with multiple confounders were analyzed using univariate log-rank tests and multiple Cox regression models that included confounders statistically significant in univariate analysis. Survival analysis was conducted in R (www.r-project.org). Statistical associations between EOR or residual tumor volumes on postoperative imaging and potential surgical and non-surgical confounders influencing tumor resectability were investigated by Spearman correlation analysis for continuous parameters and Mann–Whitney test for binary parameters. Intergroup variance was evaluated with the nonparametric Mann–Whitney test for continuous variables or Wilcoxon’s matched-pairs signed rank test for paired observations. Fisher’s exact test was used for contingency analysis using Graph-Pad Prism software (Version 5.0c, Graph Pad Inc., CA, USA).

## Results

### Patient demographics

Patient demographics including clinical and molecular parameters are listed in Table 1. Median age at first diagnosis was 35 years (range 17–54 years) with a balanced distribution between sexes. IDH1 mutations were present in 38/46 cases (83 %). IDH1 mt and IDH1 wt patients did not differ with regard to demographic-, tumor- and treatment-related factors except for their respective survival endpoints (Suppl. Table 1). Seizures were the most common presenting symptom in 65 % of patients. Median time from first imaging diagnosis to surgery was less than 1 month (range 0–91 m). Patients were followed up for a median of 70.3 months with 11 deaths (24 %) recorded meanwhile. More than half of the patients (n = 26; 57 %) experienced tumor recurrence or progression and 19 patients (41 %) suffered from malignant tumor progression. Both consecutive events were recorded in 7 patients. Survival data (OS, PFS, MPFS, TTR) are summarized in Table 1.

**Table 1:** Patient demographics

n = 46 patients	n	%
Age at first diagnosis (years; median, range)	35 (17–54)	
Sex (female:male)	25:21	54.3:45.7
IDH1 mutation	38	82.6
Follow-up (months; median, range)	69 (17.5–164.6)	
OS (months; median, range)	119.8 (17.5–164.6)	
PFS (months; median, range)	45.1 (4.7–164.6)	
MPFS (months; median, range)	81.4 (4.7–164.6)	
TTR (months; median, range)	40.9 (4.5–164.6)	
Progression	26	56.5
Malignant progression	19	41.3
Death	11	23.9
Seizure as first diagnosis	30	65.2
Time from radiographic diagnosis to surgery (months; median, range)	0 (0–91)	
KPS pre-op (median, range)	100 (80–100)	
KPS post-op (median, range)	100 (70–100)	
New permanent neurologic deficits		
None	43	93.5
Yes	3	6.5
Tumor eloquence	6	13
Tumor side (left:right)	20:26	43.5:56.5
Tumor localization (lobe)		
Frontal	23	50
Temporal	18	39.1
Others	5	10.9
Contrast enhancement pre-op	14	30.4
dT2T1 (cm^3^, median, range)	3.41 (−43.02 to 64.52)	
Upfront adjuvant treatment	7	15.2
Chemotherapy	2	
Radiotherapy	3	
Combined radio-/chemotherapy	2	
Complete resection planned	27	58.7
iMRI employed	33	71.7
Continued resection after iMRI	27	(81.8)
Vol. pre-op (cm^3^; median, range)	44.23 (0.78–193.04)	
Vol. iMRI (cm^3^; median, range)	4.95 (0–143.94)	
EOR iMRI (%; median, range)	69.5 (13.3–100)	
Vol. epMRI (cm^3^; median, range)	5.32 (0–113.9)	
EOR epMRI (%; median, range)	69.6 (10.9–100)	
Vol. follow-up MRI (cm^3^; median, range)	4.09 (0–167.98)	
EOR follow-up MRI (%; median, range)	90.4 (17.5–100)	

*OS* overall survival, *PFS* progression-free survival, *MPFS* malignant progression-free survival, *TTR* time to re-intervention, *KPS* Karnofsky Performance Score, *Vol* (tumor) volume, *iMRI* intraoperative MRI, *epMRI* early postoperative MRI, *EOR* extent of resection, *dT2T1* volumetric difference of signal abnormality between preoperative T2-FLAIR sequences and native T1 sequences

There was no surgical mortality observed in this cohort. KPS was unaltered postoperatively (median KPS: preoperative = 100, range 80–100; postoperative = 100, range 70–100, p = 0.24). Postoperative neurologic deficits were rare: 15 % experienced mild transient deficits that ceased until hospital discharge and 7 % suffered from mild permanent motor, sensory or speech deficits 3 months after surgery. No patient experienced permanent disabling deficits. No significant correlation between EOR and occurrence of new postoperative deficits was observed (p = 0.73; Mann–Whitney test).

Adjuvant treatment after first tumor resection was infrequent with 7 patients (15 %) receiving radiotherapy (RT): n = 3, chemotherapy (CHT): n = 2, or CHT+RT: n = 2. Median EOR in patients with adjuvant treatment after first tumor resection was significantly lower compared to the rest of the cohort (EOR 58.5 %; range 17.5–75.6 %; p = 0.025).

### Volumetric analysis of EOR and identification of factors influencing tumor resectability

**Fig. 1:**
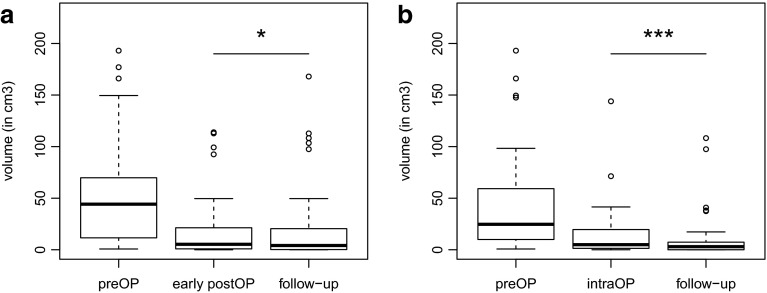
**a** Volumetric analysis of all surgically treated WHO grade II astrocytomas (n = 46). Data is presented in box-plots with medians and interquartile ranges preoperatively, on early postoperative MRI and on follow-up MRI on average 3 months after surgery. Whiskers indicate CI 95 % in cm^3^. Tumor volumes were smallest on follow-up MRI (*p < 0.05; paired Mann–Whitney test). **b** Volumetric analysis of iMRI-guided resections (n = 33). Resection was continued after iMRI in 82 % of cases yielding significantly reduced residual tumor volumes after surgery (***p = 0.0001; paired Mann–Whitney test)

Complete resection (EOR 100 %) was planned in 27 (59 %) and achieved in 10 patients (22 %), all of them being performed under iMRI guidance. In the entire cohort (n = 46), median preoperative tumor volume was 44.23 cm^3^ (range 0.78–193.04 cm^3^) and was reduced to a median residual tumor volume of 4.09 cm^3^ (range 0–167.98 cm^3^) on follow-up MRI. This corresponds to a median final EOR of 90.4 % (range 17.5–100 %) (Table 1, Suppl. Table 2; Fig. 1a). In cases of iMRI-guided surgery (n = 33), median final EOR was further increased to 94.9 % (range 34.8–100 %) and median residual volume on follow-up MRI was reduced to 2.99 cm^3^ (range 0–108.25 cm^3^) (Suppl. Table 2; Fig. 1b). Triggered by delineation of residual tumor on iMRI, additional resection was performed in most cases (27/33, 82 %). Final residual volumes and EOR were both significantly improved compared to their corresponding iMRI values (p = 0.0001 for follow-up vs. intraoperative residual tumor volume and EOR, respectively) (Suppl. Table 2; Fig. 1b). Owing to the retrospective nature of the study, allocation to iMRI-guided or conventional micro-neurosurgical resection was not controlled. EOR and residual tumor volumes were significantly influenced by patient age (EOR follow-up MRI: p = 0.03, rho = −0.34), incidental finding (Vol epMRI: p = 0.02; EOR follow-up MRI: p = 0.02; Vol follow-up MRI: p = 0.001), preoperative tumor volumes (Vol epMRI: p = 2.17E–09, rho = 0.85; EOR follow-up MRI: p = 0.0001, rho = −0.57; Vol follow-up MRI: p = 2.09E–10, rho = 0.82) and iMRI-guided surgery (EOR epMRI: p = 0.03; Vol epMRI: p = 0.01; EOR follow-up MRI: p = 0.0005; Vol follow-up MRI: p = 0.007) (Table 2). Preoperative KPS, IDH1 mutation status, preoperative dT2T1, preoperative contrast enhancement (CE) and tumor location (eloquence, side, lobe) did not impact on tumor resectability. In order to rule out that infiltrative growth differs between IDH1 mt and IDH1 wt tumors, we also investigated a possible association between IDH1 and dT2T1 as an imaging surrogate of infiltrative growth, but found none (p = 0.8; Mann–Whitney test; Suppl. Table 1).

**Table 2:** Confounders of resectability

	Volume epMRI		Volume follow-up MRI		EOR epMRI		EOR follow-up MRI	
	Spearman rho	p value	Spearman rho	p value	Spearman rho	p value	Spearman rho	p value
Continuous variables								
Age	0.162	0.367	0.264	0.087	−0.184	0.314	−0.338	**0.031**
Volume preoperative	0.845	<**0.0001**	0.818	<**0.0001**	−0.338	0.063	−0.572	<**0.001**
dT2T1 preoperative	0.269	0.143	0.159	0.334	−0.008	0.966	−0.057	0.731
Binary variables								
KPS preoperative		0.870		0.683		0.917		0.700
Incidental finding		**0.024**		**0.001**		0.555		**0.019**
Tumor eloquence		0.940		0.659		0.917		0.642
IDH1 mutation		0.314		0.208		0.397		0.577
iMRI surgery		**0.013**		**0.008**		**0.0318**		**0.001**

Analysis of confounders of resectability. Association of continuous variables was assessed by spearman correlation analysis, binary variables were analyzed with Mann–Whitney tests. Significant values are presented in bold face

*EOR* extent of resection, *dT2T1* difference in tumor volume expansion on preoperative T2 and T1 sequences, *iMRI* intraoperative MRI, *KPS* Karnofsky Performance Scale

### Confounders of overall survival

Analyzing the entire cohort of WHO grade II astrocytomas (n = 46), a universal survival advantage by extensive tumor resections was not observed. Neither EOR, nor residual tumor volumes on early postoperative or follow-up MRI were significantly associated with OS (Table 2). Likewise, stratification for EOR thresholds and dichotomization for complete (EOR 100 %) and incomplete (EOR <100 %) resections each failed to identify a prognostic benefit. To exclude a molecular bias, IDH1 mt patients were analyzed separately (n = 38). Results coincided however, showing no beneficial effect upon OS accountable to surgery (Table 3).

**Table 3:** Univariate analysis of overall survival

	Full study sample (n = 46)			IDH1 mt. (n = 38)			EOR ≥40 % (n = 39)			EOR ≥40 %/IDH1 mt. (n = 32)		
	HR	95 % CI	p value	HR	95 % CI	p value	HR	95 % CI	p value	HR	95 % CI	p value
IDH1 mutation (y/n)	**0.11**	0.03	**0.00029**		N.A.		0.21	0.03	0.058		N.A.	
		0.46						1.26				
Vol preoperative (cm^3^)	1.0073	0.9958	0.203	1.0151	0.9997	0.032	**1.0265**	1.0038	**0.005**	1.0727	0.9800	0.0005
		1.0189			1.0308			1.0497			1.1742	
Vol epMRI (cm^3^)	1.0136	0.9974	0.078	1.0201	0.9981	0.035	1.0311	0.9711	0.296	1.0351	0.9387	0.458
		1.030			1.0425			1.0948			1.1415	
Vol follow-up MRI (cm^3^)	1.0059	0.9951	0.278	1.0085	0.9966	0.148	**1.0776**	1.0116	**0.007**	**1.0925**	1.0042	**0.011**
		1.0168			1.0206			1.1480			1.1886	
EOR epMRI (%)	0.138	0.009	0.141	0.062	0.001	0.114	0.448	0.010	0.674	1.070	0.001	0.985
		2.151			2.711			19.384			1333.103	
EOR follow-up MRI (%)	0.279	0.058	0.100	0.274	0.042	0.158	0.350	0.003	0.662	0.509	0.001	0.830
		1.342			1.771			39.699			242.487	
dT2T1 preoperative (cm^3^)	0.9995	0.9635	0.980	0.9954	0.9535	0.835	**1.2170**	1.0321	**0.00009**	1.3150	0.9441	0.00005
		1.0370			1.0392			1.4349			1.8316	
Adjuvant therapy at first diagnosis (y/n)	**6.25**	1.79	**0.0010**	12.25	2.22	0.0003	4.87	0.43	0.159	12.55	0.78	0.022

p values were calculated using a log-rank test. Variables were only regarded significant if 1 was not included in 95 % confidence intervals. Significant values are presented in bold face. Volumetric measures were analyzed as un-dichotomized, continuous variables

*EOR* extent of resection, *dT2T1* difference in tumor volume expansion on preoperative T2 and T1 sequences

**Fig. 2:**
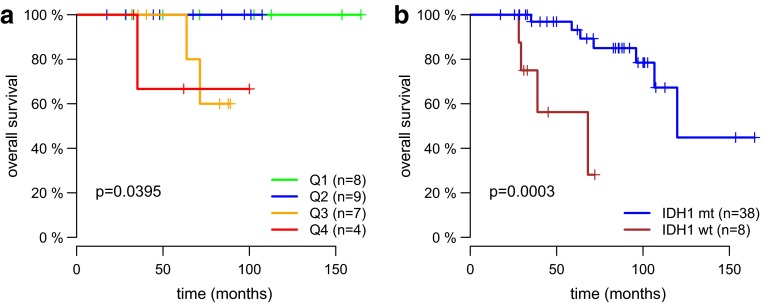
**a** Kaplan–Meier plot for OS in the subgroup of IDH1 mt patients with ≥40 %EOR. Events occur only in the bottom quartiles of patients with largest residual tumor volumes after surgery (Q1 = smallest residual volumes, Q4 = largest residual volumes on follow-up MRI) (HR 4.2; 95 % CI 0.9–19.62; p = 0.0395). **b** Kaplan–Meier plot depicting OS stratified for IDH1 mutations in WHO grade II astrocytomas. IDH1 mt patients (*blue line*) had significantly superior OS compared to IDH1 wt patients (*red line*) (HR 0.09; 95 % CI 0.02–0.42; p = 0.002)

In contrast, excluding patients with <40 % EOR (which we considered “open biopsies” rather than “tumor resections”) (Suppl. Fig. 1) revealed a strikingly different picture: In this subgroup (n = 39) smaller preoperative (HR 1.03; p = 0.005) and follow-up residual (HR 1.08; p = 0.007) tumor volumes positively impacted on OS. Notably, this effect was maintained when only IDH1 mt patients with ≥40 % EOR (n = 32) were analyzed (HR 1.09; p = 0.01 for follow-up residual volume). The Kaplan–Meier plot in Fig. 2a depicts a stepwise prolongation of OS through reduced postoperative tumor burden. In the latter subgroup (≥40 % EOR/IDH1 mt), deaths were registered only in the bottom quartiles of patients with largest residual tumor volume after surgery (p = 0.0395).

The anticipated prognostic impact of IDH1 mutation status was affirmed by a prolongation of OS in IDH1 mt patients in multivariate analysis (HR 0.09; 95 % CI 0.02–0.42; p = 0.002, Suppl. Table 3; Fig. 2b). Interestingly, adjuvant treatment at first histological diagnosis was an independent prognosticator as well and was associated with inferior OS (HR 7.13; 95 % CI 1.92–26.52; p = 0.003, Suppl. Table 3).

### Confounders of (malignant) progression-free survival and time to re-intervention

In contrast to OS, IDH1 mutation status did not impact on PFS, MPFS and TTR. However, these outcome parameters were significantly affected by extensive surgery. With respect to follow-up MRI, EOR was prognostic for PFS (HR 0.23; p = 0.031) and TTR (HR 0.23; p = 0.03), with a complete resection (EOR 100 %) also being prognostic for a superior TTR (HR 0.28; p = 0.029) (Table 3). In IDH1 mt patients, TTR was increased along with smaller residual tumor volumes at follow-up MRI (HR 1.01; p = 0.03). dT2T1 was another prognosticator of PFS (HR 1.03; p = 0.028), a finding that was most pronounced in the subgroup of resectable tumors (≥40 % EOR/IDH1 mt) (Table 3).

## Discussion

This study sought to evaluate the prognostic impact of extensive surgery in a histologically well-defined cohort of WHO grade II astrocytomas stratified for IDH1 mutation status. From a surgical point of view, we achieved a high surgical radicality (median final EOR >90 %), in particular under iMRI guidance. In survival analysis, a greater EOR was prognostic for prolonged PFS and TTR. With respect to OS, the anticipated prognostic impact of IDH1 mutation status was confirmed while a universal beneficial effect of extensive surgery was not observed. Importantly, restricting our cohort to patients with tumor resections (≥40 % EOR) rather than open biopsies, OS was significantly prolonged along with smaller residual tumor volumes.

Current evidence is in favor of a maximized EOR in LGG surgery even though most studies rely on combined analysis of various histological subtypes regardless of their distinct genetic disposition influencing individual patient survival [2, 5, 28–31]. However, with a paradigm shift from a histopathologic towards an integrated molecular classification of gliomas, it has become mandatory to stratify for histological subtypes and molecular markers in modern outcome analyses. Our approach to exclusively analyze astrocytomas stratified for IDH1 mutation status from a consecutive LGG database reflects the effort to minimize biological confounders for surgical outcome analysis and clearly distinguishes this series from others published in literature. As a proof of concept, IDH1 mutation status was an independent prognosticator for OS in our cohort which is in accordance with current literature and underlines the fact that we analyzed a representative patient sample [16–18].

A recent analysis of 200 consecutive LGG surgical cases demonstrated that IDH1 mutation status, among other molecular markers, is independent of EOR, a finding that was affirmed by our association study as well. Unfortunately, Cordier et al. did not provide imaging data that would allow conclusions on a direct link between radiographic growth patterns (e.g. infiltrative vs. circumscribed) and the underlying molecular phenotype, ultimately translating into tumor resectability [32]. Metellus et al. found IDH1 wt tumors to exhibit a more infiltrative phenotype on MRI compared to IDH1 mt counterparts [33]. Ius et al. proposed the difference between tumor expansion on T2- and T1-weighted preoperative MRI (dT2T1) as an imaging surrogate parameter of infiltrative growth in LGG that also proved to be predictive of EOR and PFS in two recent studies [3, 24]. In our analysis, a higher dT2T1 (i.e. more infiltrative radiographic growth pattern) was negatively associated with PFS as well. However, our data provide no evidence for a significant association between dT2T1 and EOR or residual tumor volumes on the one hand and dT2T1 and IDH1 mutation status on the other hand. Even though IDH mutations are early events in the formation of LGGs, their role in gliomagenesis is not particularly linked to invasiveness of tumor cells but rather to aberrant cellular metabolism resulting in oncometabolites. Thus, the molecular phenotype underlying the prognostic impact of dT2T1 needs to be further defined [34, 35].

Instead, EOR and residual tumor volumes were significantly associated with preoperative tumor burden, incidental finding, patient age and iMRI-guided surgery. Consequently, multivariate survival analysis was adjusted for these significant confounders of resectability. The high surgical radicality observed in our iMRI subgroup should encourage the use of iMRI, considering recent multicenter data in favor of high-field iMRI for GTR in LGG surgery [31]. It has to be kept in mind, however, that the uncontrolled application of iMRI in our study does not provide evidence for superiority of iMRI over conventional surgery. Our results are also in line with previous findings that smaller preoperative tumor volumes and incidentally discovered LGGs (possibly due to smaller tumor size and non-eloquent tumor location) confer a higher EOR and are also considered prognostic factors [9, 36–39]. Indeed, in our series, preoperative tumor volumes were significantly smaller in patients with complete resections (100 % EOR; n = 10) compared to the rest of the cohort (median preoperative tumor volume: 5.6 cm^3^ (range 0.78–47.2 cm^3^) vs. 56.7 cm^3^ (range 8.7–193.04 cm^3^); p = 0.004, Mann–Whitney test) but this did not translate into a survival benefit in multivariate analysis. Thus, we cannot extrapolate from our data that the beneficial outcome associated with increased EOR is independent of tumor size as a tumor-intrinsic confounder of resectability. Much larger studies are needed to clarify this issue.

In volumetric analysis, we observed a median EOR as high as 90 % in our cohort and, consequently, anticipated a beneficial impact of extensive surgery on patient outcome. Indeed, greater EOR was a positive prognosticator of PFS and TTR. Importantly, when analyzing IDH1 mt patients only, TTR was significantly increased along with smaller residual tumor volumes. This finding offers the prospect for long-lasting disease control through surgical intervention regardless of molecular markers and might also impact on quality of life [1, 8, 40, 41]. In our cohort, a greater EOR was not at the expense of additional neurologic morbidity. It must be admitted, however, that the percentage of tumors located within eloquent regions (13 %) was rather low compared to literature [42]. Since this is a retrospective analysis, we cannot rule out that some surgeons might have been reluctant to offer extensive surgery in case of involvement of presumed eloquent areas and this has reduced the number of eloquent tumors considered for resection in our cohort. Moreover, among the inconsistent definitions of “eloquence” in the literature, leading to a highly variable percentage of “eloquent tumors”, only brain regions directly associated with motor or speech function were regarded eloquent in this study while functional areas related to e.g. sensory function and vision were disregarded.

With respect to OS, our analysis failed to observe a universal survival advantage conferred by extensive surgery, in contrast to larger surgical outcome studies [1, 2, 4]. Even though a molecular bias could be accused to override possible benefits of surgery on patient outcome, analyzing IDH mt patients only did not turn EOR or residual tumor volumes into prognosticators for OS either. This finding may be explained by compiling our patient sample based on histological rather than surgical criteria. Hence, our cohort included a broad range of preoperative tumor volumes (0.78–193.04 cm^3^) with heterogeneous tumor locations, likely resulting in divergent surgical goals that ranged from extended biopsy to complete resection and ultimately led to an EOR ranging from of 17.5 to 100 % on follow-up MRI. To minimize surgical bias caused by different surgical prerequisites, we sought to preclude all patients with extended biopsies rather than tumor resections from further survival analysis. The cut-off was set at 40 % EOR, incorporating evidence from a large volumetric LGG series that failed to demonstrate any survival benefit below a 40 % EOR cut-off [2] since our own study sample was too small to identify an EOR threshold with prognostic impact. This maneuver erased seven tumors with a high median preoperative volume of 134 cm^3^ from outcome analysis, including four of the six largest tumors in our study sample (Suppl. Fig. 1). In the remaining subgroup of patients with an EOR ≥40 % (n = 39), preoperative and follow-up MRI residual tumor volumes eventually showed a significant impact on OS. This finding supports previous studies reporting that not only relative EOR but in particular absolute residual tumor burden affects outcome [2, 9].

Interestingly, only EOR and residual tumor volumes depicted on first follow-up, but not on early postoperative MRI predicted patient outcome. Following imaging protocols for HGG, the amount of residual tumor is commonly assessed on early postoperative MRI in LGG as well, albeit any sustainable evidence [43]. However, Belhawi et al. previously illustrated how postoperative signal alterations caused by post-resection injury can particularly bias interpretation of early postoperative FLAIR imaging [44]. This leads to a systematic overestimation of residual tumor and, as a consequence, possibly underestimates the prognostic significance of extensive surgery. Since we rigorously defined GTR as 100 % EOR, or “no T2/FLAIR hyperintense residual tumor”, post resection signal alterations likely also contributed to the apparent gap between 27 planned and 10 achieved GTRs in our series, despite the use of follow-up MRI for endpoint definition. Nevertheless, median EOR was 90.4 % in our cohort, which is considerably high compared to the literature. Our finding concerning the exclusive prognostic significance of follow-up MRI is subject to further investigation.

Owing to the retrospective nature of our study, there are some limitations hindering interpretation of study results. Most importantly, our retrospective cohort was prone to surgical bias (heterogeneity of tumor size and location; a potential selection bias towards non-eloquent tumors; different surgeons’ attitude towards resection limits; the uncontrolled, albeit frequent use of iMRI) that was partly resolved by subgroup analysis for ≥40 % EOR cases and multivariate survival analysis adjusted for confounders of resectability (among those preoperative tumor volume and iMRI-guided surgery). Non-standardized and heterogeneous application of upfront adjuvant treatment after first tumor resection added another confounder to our small sample size even though this mirrors current clinical practice at the time our patients were treated. Noteworthy, adjuvant treatment at first diagnosis was an independent negative prognosticator of OS, a finding that has been described by other surgical outcome studies as well but does not allow conclusions regarding the value of upfront radio- or chemotherapy [2, 29, 31]. Nevertheless, the strength of our study design, in contrast to most other surgical outcome studies, lies in the analysis of a histology-adjusted cohort stratified for prognostic molecular markers, providing reliable and comparable information about the impact of extensive surgery in the era of molecular classification of gliomas.

## Conclusion

In WHO grade II astrocytomas, reduction of postoperative tumor burden was prognostic for OS within the subgroup of IDH1-mutated, ≥40 % EOR tumors. Moreover, extensive surgery was predictive of PFS and TTR, even when analyzing IDH1 mt patients only, which is an asset that goes beyond the aspect of survival time. Therefore, these results should question the rationale of a biopsy-driven treatment approach in resectable low-grade astrocytomas. Our approach highlights the need to analyze well-defined patient cohorts stratified for histological subtypes and molecular markers and illustrates the diverse impact of biological and surgical confounders upon patient survival.

## Electronic supplementary material

Below is the link to the electronic supplementary material.

Supplementary material 1 (DOCX 2352 KB)
