# Supplementary material for: Prognostic value of the extent of resection in supratentorial WHO grade II astrocytomas stratified for IDH1 mutation status: a single-center volumetric analysis
Source: J Neurooncol. 2016 Jun 25;129:319–28. doi: 10.1007/s11060-016-2177-y (PMC4992014; doi:10.1007/s11060-016-2177-y)
Supplement: Supplementary file 1 — Supplementary material 1 (DOCX 2352 KB) [file 11060_2016_2177_MOESM1_ESM.docx]

**Suppl. Fig. 1**





**Suppl. Fig. 1** Graphical summary of preoperative tumor volumes vs. EOR on follow-up MRI in the entire cohort of 46 WHO grade II astrocytomas. A 40% EOR cut-off (dotted line) was applied to stratify for different goals of surgery (extended biopsies vs. tumor resections) in a retrospective setting. Each data point represents one case. Solid data points denote iMRI-guided surgery.

**Suppl. Table 1 Patient Demographics of IDH *mt* and IDH *wt* patients**

|  |  | **IDH1 *mt*** | **IDH1 *wt*** |  | **p-value** |
| --- | --- | --- | --- | --- | --- |
| **n=46 patients** |  | **n=38** | **n=8** |  |  |
| **Age at 1^st^ diagnosis** (years; median, range) |  | 34 (17-53) | 38 (24-54) |  | 0.36^a^ |
| **Sex** (female:male) |  | 22: 16 | 3 : 5 |  | 0.44^b^ |
| **Follow-up** (months; median, range) |  | 83.4 (17.5-164.6) | 35.4 (27.9-72.1) |  | **0.001^c^** |
| **OS** (months; median, range) |  | 119.8 (35.2-164.6) | 68.1 (28.1-72.1) |  | **0.0003^c^** |
| **PFS** (months; median, range) |  | 43.8 (4.7-164.6) | N.A.* (8.3-72.1) |  | 0.1^c^ |
| **MPFS** (months; median, range) |  | 110.6 (4.7-164.6) | 57.5 (8.3-72.1) |  | **0.01^c^** |
| **TTR** (months; median, range) |  | 40.9 (4.5-164.6) | 35.7 (6.7-72.1) |  | 0.33**^c^** |
| **Progression** |  | 23 | 2 |  | 0.12^b^ |
| **Malignant progression** |  | 15 | 4 |  | 0.7^b^ |
| **Death** |  | 7 | 4 |  | 0.079^b^ |
| **Time from radiographic diagnosis to surgery** (months; median, range) |  | 0 (0-91) | 0 (0-17) |  | 0.63^a^ |
| **KPS pre-op** (median, range) |  | 100 (80-100) | 100 (90-100) |  | 1^a^ |
| **KPS post-op** (median, range) |  | 100 (70-100) | 100 (70-100) |  | 0.58^a^ |
| **New permanent neurologic deficits**  - None  - Yes |  | 38  0 | 8  0 |  | 0.34^b^ |
| **Tumor eloquence** |  | 5 | 1 |  | 1^b^ |
| **Tumor side** (left:right) |  | 16 : 22 | 4 : 4 |  | 0.71^b^ |
| **Tumor localization** (lobe)  - Frontal  - Temporal  - Others |  | 19  16  3 | 4  2  2 |  | 1^b^  (frontal  vs. others) |
| **Contrast enhancement pre-op** |  | 11 | 3 |  | 0.66^b^ |
| **dT2T1** (cm^3^; median, range) |  | 4.7 (0.3 – 64.5) | 3.1 (0.2 – 18.7) |  | 0.44 ^a^ |
| **Complete resection planned** |  | 22 | 5 |  | 0.68^b^ |
| **iMRI employed** |  | 29 | 4 |  | 0.2^b^ |
| **Vol pre-op** (cm^3^; median, range) |  | 48.7 (0.9 – 193) | 8.7 (1.3 – 64.2) |  | 0.06^a^ |
| **Vol iMRI** (cm^3^; median, range) |  | 4.95 (0 – 143.9) | 3.6 (0.2 – 19.6) |  | 0.64^a^ |
| **EOR iMRI** (%; median, range) |  | 68.8 (13.3 – 100) | 72.3 (64.8 – 83.8) |  | 0.85^a^ |
| **Vol epMRI** (cm^3^; median, range) |  | 5.8 (0 – 113.9) | 1.5 (0 – 35.2) |  | 0.31^a^ |
| **EOR epMRI** (%; median, range) |  | 69.6 (10.9 – 100) | 68.2 (43.1 – 100) |  | 0.40^a^ |
| **Vol follow-up MRI** (cm^3^; median, range) |  | 4.3 (0 – 168) | 0.5 (0 – 23.6) |  | 0.21^a^ |
| **EOR follow-up MRI** (%; median, range) |  | 89.9 (20.5 – 100) | 92.6 (17.5 – 100) |  | 0.58^a^ |
| **Adjuvant therapy after 1^st^ diagnosis** |  | 5 | 2 |  | 0.59^b^ |
| **Chemotherapy** at progression |  | 18 | 4 |  | 1^b^ |
| **Radiotherapy** at progression |  | 18 | 3 |  | 0.71^b^ |
| **No. of resections**  - 1  - 2  - 3 |  | 26  9  3 | 6  2  0 |  | 1^b^  (1 vs. 2,3) |

OS: overall survival; PFS: progression-free survival; MPFS: malignant progression-free survival; TTR: time to re-intervention; KPS: Karnofsky Performance Score; Vol: (tumor) volume; iMRI: intraoperative MRI; epMRI: early postoperative MRI; EOR: extent of resection dT2T1: Volumetric difference of signal abnormality between preoperative T2-FLAIR sequences and native T1 sequences. *Note that median PFS was not available (N.A.) for IDH1 *wt* patients due to the low number of events.

**^a^** Mann-Whitney test, **^b^** Fisher’s exact test, ^c^ log-rank test; Significant values (p<0.05) are presented in **bold face**.

**Suppl. Table 2 Volumetric Analysis**

|  |  | **Full study sample** | **iMR surgery** | |
| --- | --- | --- | --- | --- |
| Number of patients (n) |  | **46** | **33** | |
| **Tumor Volumes** |  | | |  |
| **Vol preoperatively** (cm^3^; median, range) |  | 44.23 (0.78 – 193.04) | 24.69 (0.78 – 193.04) | |
| **Vol iMRI** (cm^3^; median, range) |  | NA | 4.95 (0 – 143.94) | |
| **Vol epMRI** (cm^3^; median, range) |  | 5.32 (0 – 113.9) | 3.42 (0 – 99.25) | |
| **Vol follow-up MRI** (cm^3^; median, range) |  | 4.09 (0 – 167.98) | 2.99 (0 – 108.25) | |
| **EOR** |  | | |  |
| **EOR iMRI** (%; median, range) |  | NA | 69.5 (13.3 – 100) | |
| **EOR epMRI** (cm^3^; median, range) |  | 69.6 (10.9 – 100) | 81.2 (10.9 – 100) | |
| **EOR follow-up MRI** (%; median, range) |  | 90.4 (17.5 – 100) | 94.9 (34.8 – 100) | |
| **EOR: Smith classification^1^** (n; %)  - 100%  - 90-99%  - 41-89%  - < 40%  - NA |  | 10 (21.7)  11 (23.9)  14 (30.4)  7 (15.2)  4 (8.8) | 10 (30.3)  10 (30.3)  9 (27.4)  2 (6.0)  2 (6.0) | |

Vol: (tumor) volume; EOR: extent of resection; iMRI: intraoperative MRI; epMRI: early postoperative MRI

**^1^**Allocation of patients according to EOR as performed by [2].

**Suppl. Table 3 Multivariate Analysis for Confounders of Survival**

|  |  |  |  |  | |
| --- | --- | --- | --- | --- | --- |
| **OS** | **HR** | **95% C.I** | **p-value** | |  |
| IDH1 mutation | 0.091 | (0.020 – 1.918) | **0.002** | |  |
| Adjuvant therapy at 1^st^ diagnosis | 7.132 | (1.918 – 26.522) |  | **0.003** |  |
|  |  |  |  |  |  |
| **PFS** |  |  |  |  |  |
| EOR follow-up MRI | 0.302 | (0.070 – 1.30) | 0.108 | |  |
| dT2T1 preoperative | 1.03 | (1.0 – 1.056) | 0.066 | |  |
|  |  |  |  |  |  |
| **MPFS** |  |  |  |  |  |
| N.A. | N.A. |  |  | |  |
|  |  |  |  |  |  |
| **TTR** |  |  |  |  |  |
| 100% EOR | 0.338 | (0.083 – 1.374) | 0.13 | |  |
| EOR follow-up MRI | 0.492 | (0.092 – 2.623) | 0.406 | |  |
|  |  |  |  |  |  |

Multivariate regression models for different study endpoints. Confounders significant in the univariate analysis were included. Due to a low number of events, a maximum of 2 confounders per model was feasible. OS: overall survival; PFS: progression-free survival; MPFS: malignant progression-free survival; TTR: time to re-intervention. EOR: extent of resection, dT2T1: difference in tumor volume expansion on preoperative T2 and T1 sequences. Significant values are presented in **bold face**. Note that no significant parameters were obtained for MPFS (N.A. = not applicable).
